# Psychological Outcomes and Quality of Life of Fibromyalgia Patients with Vitamin D Supplementation—A Meta-Analysis

**DOI:** 10.3390/jcm12072750

**Published:** 2023-04-06

**Authors:** Chia-Chun Yang, Sheng-Ta Tsai, Berne Ting, Ying-Chih Cheng, Chin-Kun Wang, Jane Pei-Chen Chang, Kuan-Pin Su

**Affiliations:** 1 Department of General Psychiatry, Taoyuan Psychiatric Center, Taoyuan 330, Taiwan; chungang249@gmail.com; 2 Mind-Body Interface Laboratory (MBI-Lab), Department of Psychiatry, China Medical University Hospital, Taichung 404, Taiwan; berne.ting@gmail.com; 3 School of Medicine, China Medical University, Taichung 404, Taiwan; tshengdar@gmail.com; 4 Department of Neurology, China Medical University Hospital, Taichung 404, Taiwan; 5 Neuroscience and Brain Disease Center, China Medical University, Taichung 404, Taiwan; 6 Ph.D. Program for Aging, College of Medicine, China Medical University, Taichung 404, Taiwan; 7 Department of Psychiatry, China Medical University Hsinchu Hospital, China Medical University, Hsinchu 302, Taiwan; b101091022@gmail.com; 8 Institute of Epidemiology and Preventive Medicine, College of Public Health, National Taiwan University, Taipei 106, Taiwan; 9 Research Center of Big Data and Meta-Analysis, Wan Fang Hospital, Taipei Medical University, Taipei 110, Taiwan; 10 Department of Nutrition, Chung Shan Medical University, Taichung 402, Taiwan; wck@csmu.edu.tw; 11 Graduate Institute of Biomedical Sciences, China Medical University, Taichung 404, Taiwan; 12 An-Nan Hospital, China Medical University, Tainan 709, Taiwan

**Keywords:** vitamin D, fibromyalgia, quality of life, psychological outcomes, nutrition

## Abstract

The efficacy of current pharmaceutical treatments for fibromyalgia are limited. Vitamin D has shown promise in relieving pain. However, there is a lack of comprehensive analysis of psychological outcomes with vitamin D supplementation in fibromyalgia. This study aimed to investigate the impact of vitamin D supplementation on psychological outcomes and quality of life in fibromyalgia patients, given the unmet clinical need for effective treatment options. A meta-analysis of randomized controlled trials comparing vitamin D to placebo and prospective studies examining changes before and after vitamin D supplementation for patients with fibromyalgia was conducted to evaluate the effects of vitamin D on psychological outcomes, quality of life, and pain scores in patients with fibromyalgia. Databases were searched for relevant articles published from earliest available date to October 31, 2022. (PROSPERO number, CRD42022369889). We included 8 trials with a total of 694 participants and found that vitamin D supplementation had significant positive effects on physical function (standard mean differences (SMD) = 0.44, 95% CI = [0.10, 0.77 ]), role limitations due to emotional health (SMD = 0.57, 95% CI = [0.32, 0.82]), social function (SMD = 0.50, 95% CI = [0.08, 0.93]), and general health (SMD = 0.36, 95% CI = [0.11, 0.61]). Improvement of the Fibromyalgia Impact Questionnaire (FIQ) scores was noted (SMD = −0.414, 95% CI = [−0.808, −0.021]), but not on the Visual Analog Scale (VAS) (SMD = −0.15, 95% CI = [−0.771, 0.471]) and the Beck’s Depression Inventory (BDI) scores (SMD = −0.456, 95% CI = [−1.27, 0.30]). In conclusion, vitamin D supplementation might be an alternative option for improvement of psychological outcomes and quality of life in patients with fibromyalgia.

## 1. Introduction

Fibromyalgia is a complex syndrome of unknown origin with multiple causative factors. It is characterized by chronic and widespread pain, fatigue, unrefreshing sleep, cognitive dysfunction, depression, and other somatic symptoms such as muscle weakness, headaches, abdominal pain/cramps, numbness/tingling, dizziness, and insomnia [1]. (The estimated prevalence of fibromyalgia in the general population ranges from 2 to 4%, with a higher incidence in women compared to men [1,2,3]. Patients with fibromyalgia are associated with a higher annual medical service costs, resulting in significant health care and economic burden [4,5]. The management of fibromyalgia is typically multimodal, encompassing patient education, fitness programs, pharmacotherapy, and psychotherapy tailored to the individualized symptoms of each patient [6]. Nevertheless, a mere 25% of fibromyalgia patients reported long-term improvement following treatment [7]. There exists a substantial unmet medical need for the treatment of patients with fibromyalgia (FMS).

Mounting evidence suggests the potential benefits of nutritional supplements in managing inflammatory conditions such as depression [8,9] and somatic symptoms of fibromyalgia [10,11]. Vitamin D, an essential steroid derivative with neuroactive properties, plays a crucial role in central and peripheral nervous system physiology [12]. Vitamin D deficiency has been implicated in pain behavior and subsequent alterations in spinal cord sensory neuron activity [13,14,15]. Furthermore, vitamin D is involved in the bidirectional relationship between pain and sleep [16] and has been shown to reduce negative emotions in patients with major depressive disorder [17]. Notably, patients with fibromyalgia have significantly lower levels of serum vitamin D compared to those without the condition [15,18].

Vitamin D and its receptor modulate pain by regulating the genes critical in pain signaling [19]. Vitamin D supplementation has been shown to improve emotional anxiety symptoms in patients with major depressive disorder [17,20,21] and relieve post-herpetic neuralgia by enhancing antiviral efficacy [22]. Vitamin D deficiency has been associated with the severity of inflammatory bowel disease [23,24], and it plays a crucial role in mitigating inflammation by deactivating the nuclear factor kappa-light-chain-enhancer of activated B cells (NF-κB) signaling pathway [25]. Fibromyalgia is associated with acute or chronic tissue injuries that lead to the release of inflammatory cytokines, resulting in the long-term activation of dorsal horn neurons and spinal cord glia and central sensitization [26,27,28]. Therefore, the anti-inflammatory properties of vitamin D make it a potential therapeutic option for individuals with fibromyalgia.

A recent meta-analysis has shown that vitamin D supplementation can reduce the Fibromyalgia Impact Questionnaire (FIQ) scores in fibromyalgia patients, although no significant difference was observed in Visual Analogue Scale (VAS) scores [29]. However, the impact of vitamin D supplementation focusing on the psychological outcomes of fibromyalgia has not been extensively studied. Thus, we conducted a meta-analysis to investigate whether vitamin D supplementation can improve psychological well-being and quality of life in patients with fibromyalgia.

## 2. Materials and Methods

### 2.1. Data Sources and Search Strategy

This systematic review and meta-analysis was conducted following the guidelines of the Preferred Reporting Items for Systematic Reviews and Meta-Analyses (PRISMA) statement [30]. Relevant studies were identified through a computerized search of PubMed, the Cochrane Library, EMBASE, and Web of Science from their inception to October 2022, using key MeSH terms of ((Vitamin D) AND (fibromyalgia)), ((Vitamin D) AND (chronic widespread pain)), ((Vitamin D) AND (chronic musculoskeletal pain)), ((Vitamin D) AND (FMS)). The literature search was conducted by two researchers (C.-C. Yang, with five years of clinical practice, and S.-T. Tsai, with ten years of clinical practice). We searched keywords, text, titles, and subject headings for each database. All publications with titles meeting the inclusion criteria were reviewed, and randomized control trials (RCT) and cohort studies investigating the effects of vitamin D supplementation on fibromyalgia were eligible for inclusion. Additionally, we searched the reference lists of primary included articles to identify any additional relevant studies.

### 2.2. Inclusion and Exclusion Criteria

Our objective was to assess the impact of vitamin D supplementation on the psychological well-being of individuals with fibromyalgia. Eligible studies had to meet the following criteria: (a) randomized controlled trials or prospective cohort studies; (b) enrollment of fibromyalgia patients who received vitamin D supplementation; (c) reporting of psychological outcomes and quality of life, measured by the 36-item Short Form Health Survey (SF-36), FIQ, Beck Depression Inventory (BDI), VAS, Widespread pain index (WPI), or Pittsburgh Sleep Quality Index (PSQI); and (d) published in English or Chinese. Articles reporting a case or series of cases, review articles, animal studies, conference abstracts, editorials, and letters were excluded (Supplementary Table S2, [4,5,7,10,14,18,25,29,31,32,33,34,35,36,37,38,39,40,41,42,43,44,45,45,46,47,48,49,50,51,52,53,54,55,56,57,58,59,60,61,62,63,64,65,66,67,68,69,70,71,72,73,74,75,76,77,78,79,80,81,82,83,84,85,86,87,88,89]). For randomized controlled trials, we compared the effect of vitamin D supplementation or placebo on fibromyalgia patients. For prospective cohort studies, we compared the psychological outcomes and quality of life before and after vitamin D supplementation in the same group of fibromyalgia patients.

### 2.3. Data Extraction and Quality Appraisal

Two reviewers, C.-C. Yang and S.-T. Tsai, independently extracted data on the authors, publication year, study design, sample sizes, age, geographical location, duration and dosage of vitamin D supplementation, and baseline and endpoint psychological outcome and quality of life scores from the included articles. The means and standard deviations of changes from baseline were also recorded. Any discrepancies between the reviewers were resolved by a third reviewer, B. Ting. The Cochrane Collaboration risk of bias tool was used to assess the methodological quality of the included RCTs [90], while the Newcastle–Ottawa Scale was used to evaluate the quality of the included prospective cohort studies [91]. Any discrepancies between the two reviewers were discussed and resolved.

### 2.4. Outcomes Assessments

The present study included both randomized controlled trials (RCTs) and prospective studies, which were analyzed separately. The primary outcome of interest was the change in SF-36 scores before and after vitamin D supplementation in the included prospective studies. The secondary outcomes were the standard mean differences of FIQ, VAS, and BDI between the vitamin D group and reference (placebo) group without vitamin D supplementation, for the included RCTs.

### 2.5. Statistical Analysis

The statistical analysis was carried out using version 3 of Comprehensive Meta-Analysis (CMA) software. A *p*-value less than 0.05 was considered statistically significant. The standard deviations (SDs) were estimated using reported confidence interval (CI) limits, standard error, or range values. The precision of an effect size was determined by calculating the 95% CI. Initially, a random effects model was employed to pool individual standard mean differences (SMDs) given the high heterogeneity assumed among the included studies. The degree of statistical heterogeneity was assessed using the *I*^2^ and Cochrane’s Q test, with *I*^2^ quantifying the proportion of the total outcome variability that was due to variability among the studies.

## 3. Results

### 3.1. Baseline Characteristics of Included Studies

**Figure 1:**
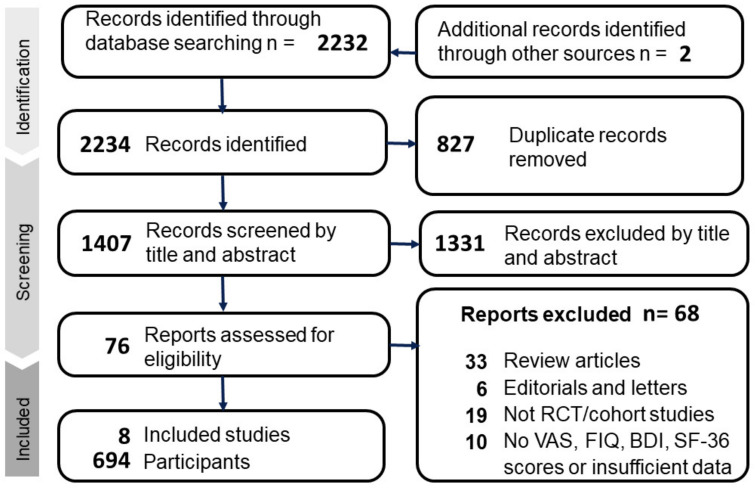
Flow chart of the selection strategy and inclusion and exclusion criteria.

In the initial database search, a total of 2234 records were identified, which was reduced to 1407 after removing duplicates. After screening for relevance, eight articles met the inclusion criteria, as shown in Figure 1. The included articles were conducted in America [31,92], Austria [93], Iran [94], Mexico [95], Norway [96], and Turkey [97,98]. Table 1 presents a summary of the characteristics of the included articles. In relation to the diagnostic criteria for fibromyalgia, the utilization of various methodologies has been observed across six relevant studies. Specifically, two of these studies employed the diagnostic criteria proposed by the American College of Rheumatology (ACR) in 1990 [99], two others utilized the revised ACR criteria introduced in 2010 [100], two studies employed either the 1990 or 2010 ACR criteria, and the remaining two studies did not provide information on the specific diagnostic criteria utilized. Additionally, all studies included in our analysis excluded patients with pre-existing psychiatric disorders. The mean age of participants ranged from 36 to 59.3, with sample sizes ranging from 30 to 215. Most studies administered a weekly dosage of 50,000 IU of vitamin D, except for one RCT conducted by Knutsen et al. [96], which administered 25 micrograms (1000 IU) daily. The duration of intervention ranged from 8 to 20 weeks (Table 1).

**Figure 2:**
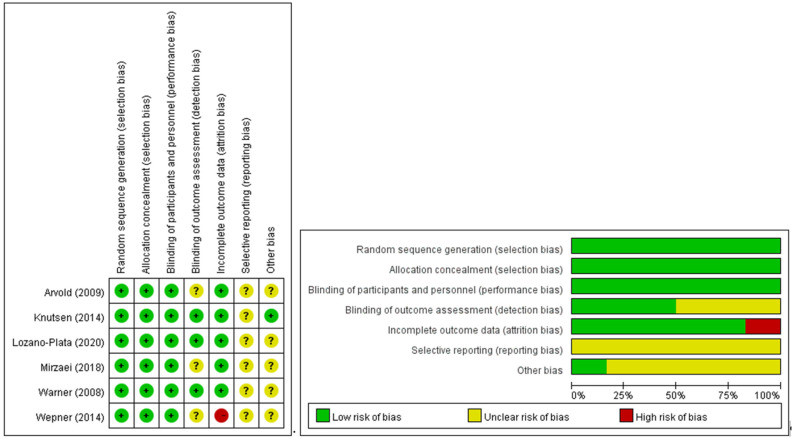
Risk of bias for six RCT studies. Left: Risk of bias summary: review authors’ judgements about each risk of bias item for each included study. Green circle: low risk of bias; yellow circle: unclear risk of bias; red circle: high risk of bias. Right: Risk of bias graph: review authors’ judgements about each risk of bias item presented as percentages across all included studies [31,92,93,94,95,96].

**Figure 3:**
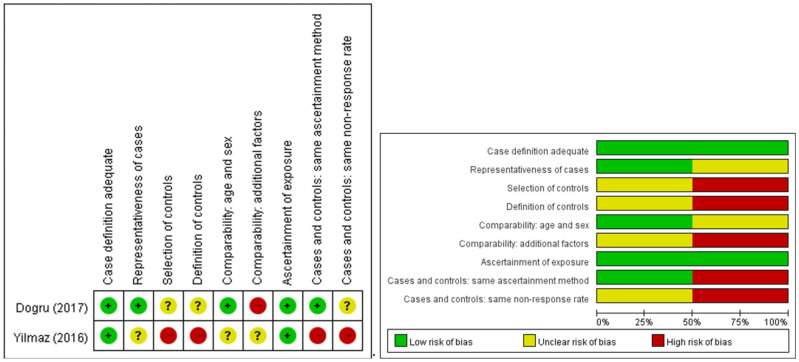
Risk of bias for the two non-randomized studies. Left: Risk of bias summary: review authors’ judgements about each risk of bias item for each included study. Green circle: low risk of bias; yellow circle: unclear risk of bias; red circle: high risk of bias. Right: Risk of bias graph: review authors’ judgements about each risk of bias item presented as percentages across all included studies [97,98].

The majority of investigations were conducted as randomized controlled trials, with two exceptions that followed an uncontrolled prospective cohort design. A depiction of the methodological rigor of the encompassed studies is provided in Figure 2 and Figure 3.

### 3.2. Psychological Effects of Vitamin D Supplementation on Fibromyalgia

**Figure 4:**
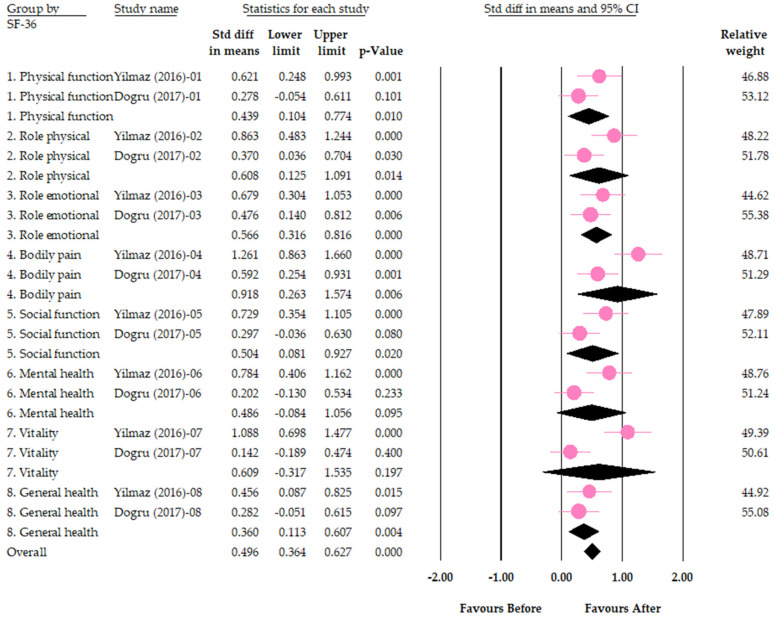
Forest plot for the effects of vitamin D on psychological outcomes (measured by domains of SF-36 questionnaire). Role physical = role limitations due to physical health problems; Role emotional = role limitations due to emotional health problems. The pink circles indicate the result of each study. The black diamonds indicate the pooled result of the two studies [97,98].

In two separate prospective cohort studies, the efficacy of vitamin D supplementation was evaluated by examining the improvement of each of the eight domains of SF-36 after a 12-week intervention [97,98]. A meta-analysis of the pooled results revealed that four of the eight domains exhibited significant improvement subsequent to vitamin D supplementation, namely physical function (SMD = 0.44, 95% CI = [0.10, 0.77]), role limitations due to emotional health problems (Role emotional) (SMD = 0.57, 95% CI = [0.32, 0.82]), social function (SMD = 0.50, 95% CI = [0.08, 0.93]), and general health (SMD = 0.36, 95% CI = [0.11, 0.61]). Conversely, no notable improvement was observed in four other domains, including role limitations due to physical health problems (Role physical) (SMD = 0.61, 95% CI = [0.13, 1.09]), bodily pain (SMD = 0.92, 95% CI = [0.26, 1.57]), mental health (SMD = 0.49, 95% CI = [−0.09, 1.06]), and vitality (SMD = 0.61, 95% CI = [−0.32, 1.53]) (Figure 4). With respect to heterogeneity, significant heterogeneity was not observed in physical function (I^2^ = 40%, *p* for I^2^ = 0.20), role limitations due to emotional health problems (Role emotional) (I^2^ = 0%, *p* = 0.46), social function (I^2^ = 61%, *p* = 0.11), and general health (I^2^ = 0%, *p* = 0.50), while significant heterogeneity was observed in the domains of role limitations due to physical health problems (Role physical) (I^2^ = 68%, *p* = 0.08), bodily pain (I^2^ = 79%, *p* = 0.03), mental health (I^2^ = 78%, *p* = 0.03), and vitality ((I^2^ = 90%, *p* = 0.001).

For SF-36 1. Physical function: I^2^ = 40%; For SF-36 2. Role physical: I^2^ = 68%; For SF-36 3. Role emotional: I^2^ = 0%; For SF-36 4. Bodily pain: I^2^ = 79%; For SF-36 5. Social function: I^2^ = 61%; For SF-36 6. Mental health: I^2^ = 78%; For SF-36 7. Vitality: I^2^ = 91%; For SF-36 8. General health: I^2^ = 0%

**Figure 5:**
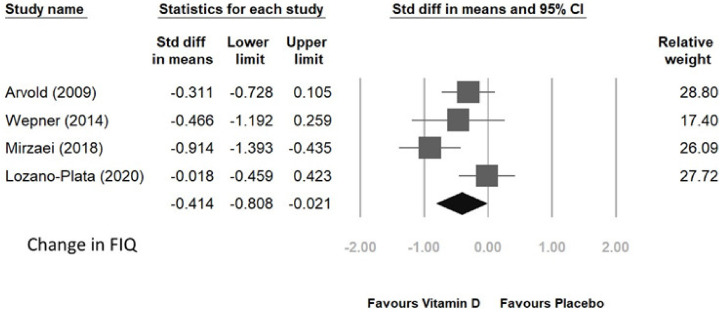
Change in FIQ score after vitamin D supplement [92,93,94,95].

**Figure 6:**
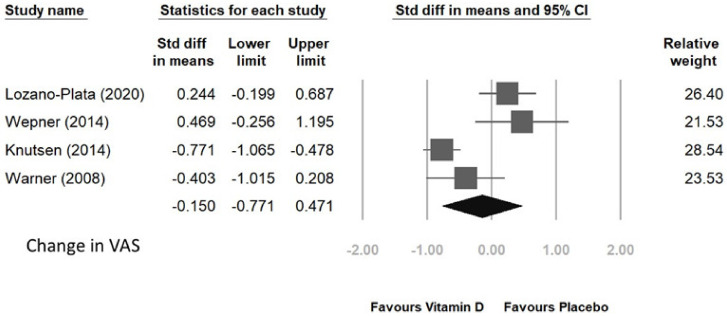
Change in VAS after vitamin D supplement [31,93,95,96].

Four randomized controlled trials were conducted to analyze the FIQ and VAS scores in patients who received vitamin D supplementation compared to those who received placebo. The supplementation period ranged from 8 to 20 weeks. The pooled results of the four trials demonstrated a significant improvement in FIQ score with a standardized mean difference (SMD) of −0.414 and 95% CI of [−0.808, −0.021], as shown in Figure 5. However, no significant improvement was observed in VAS score, with an SMD of −0.15 and 95% CI of [−0.771, 0.471] (Figure 6).

Heterogeneity was observed in FIQ score (I^2^ = 60%, *p* = 0.06). In contrast, no heterogeneity was observed in VAS changes (I^2^ = 85%, *p* = 0.63).

**Figure 7:**
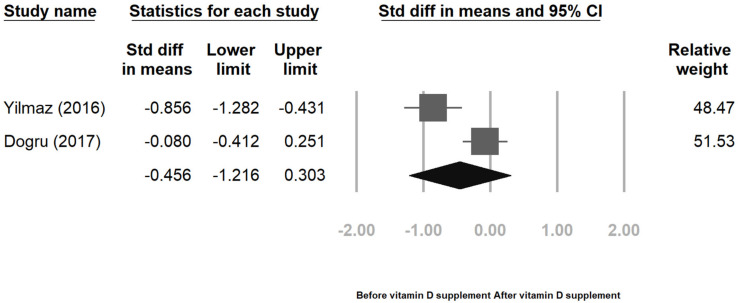
Change in BDI after vitamin D supplement [97,98].

Two prospective cohort studies analyzed the BDI score; the pooled result of these two studies showed non-significant (SMD = −0.456, 95% CI = [−1.27, 0.30]) (Figure 7). Although these two studies exhibited heterogeneity (I^2^ = 87%, *p* = 0.005), the heterogeneity might be biased as the number of studies is small [101].

## 4. Discussion

To our knowledge, this is the first meta-analysis focused on psychological outcomes of vitamin D supplementation in patients with fibromyalgia. The analysis performed in this investigation yields the following outcomes: (a) prospective cohort studies reveal that vitamin D supplementation enhances the physical function, role limitations due to emotional health problems, social function, and general health of patients suffering from fibromyalgia and (b) randomized controlled trials demonstrate that vitamin D diminishes the scores on the FIQ of individuals afflicted with fibromyalgia.

What is the operational definition of psychological outcomes in fibromyalgia? As is commonly known, fibromyalgia is a complex syndrome that causes patients to suffer from a variety of symptoms beyond pain, including fatigue, poor sleep, cognitive dysfunction, depression, and others. These symptoms can significantly reduce the quality of life of patients and may even contribute to pain sensations [102]. To better understand the psychological outcomes associated with fibromyalgia, our research team specializing in psychology extracted data from six standardized instruments, including the 36-item Short Form Health Survey (SF-36), Fibromyalgia Impact Questionnaire (FIQ), Beck Depression Inventory (BDI), Visual Analog Scale (VAS), Widespread Pain Index (WPI), and Pittsburgh Sleep Quality Index (PSQI). The primary objective of this meta-analysis was to assess the psychological outcomes of vitamin D supplementation, utilizing the Medical Outcomes Study 36-Item Short Form (SF-36). The SF-36 survey is a validated and reliable measure of general health-based quality of life, widely implemented across medical disciplines which include physical function, social function, role limitations due to physical and emotional problems (two distinct items), mental health, vitality, bodily pain, and general health perception [103]. Item scores for each dimension range from 0 to 100, signifying the poorest and the best status, respectively [104]. A prospective study reported substantial enhancement across all domains of SF-36 subsequent to 12 weeks of vitamin D supplementation [97]. Another study reported significant improvements in seven out of eight SF-36 domains, with the exception of bodily pain, before and after vitamin D therapy [98]. Currently, there is a lack of randomized controlled trials that examine the SF-36 outcomes in patients with fibromyalgia.

The secondary finding is that vitamin D supplementation had positive effects on Fibromyalgia Impact Questionnaire (FIQ) scores in patients with fibromyalgia. The FIQ is a self-administered tool used to assess the health status of fibromyalgia patients, which measures physical function, work status, depression, anxiety, morning tiredness, stiffness, pain, fatigue, and well-being over the past week. A previous meta-analysis had reported positive effects of vitamin D supplementation on FIQ scores in fibromyalgia patients [29]. In the current studies, two RCTs reported significant improvement in all subscores of FIQ in patients treated with vitamin D, as compared to those without vitamin D supplementation [92,94]. Another RCT showed slight improvements in total FIQ scores in both vitamin D and placebo groups, but a significantly better outcome in morning fatigue in the vitamin D group [93]. However, a recent RCT conducted in Mexico did not observe any improvement in FIQ after 12 weeks of vitamin D supplementation [95]. This inconsistency in results may be due to differences in sample size, severity of fibromyalgia (different baseline FIQ), or confounding variables in these nutritional intervention studies [69].

In our meta-analysis, we observed no significant improvement in VAS and BDI scores following vitamin D supplementation. While one RCT reported a reduction in VAS scores [96], the remaining three RCTs did not observe any significant change in VAS scores with vitamin D supplementation [31,93,95]. These findings were consistent with a previous meta-analysis which suggested that vitamin D supplementation could improve pain and decrease pain scores despite no significant change in VAS [32]. In terms of BDI, one prospective study found a marked decrease in BDI scores following vitamin D supplementation for 12 weeks [97], while another study failed to replicate this finding [98]. Some potential confounding factors exist since the assessment tools for fibromyalgia are patient-dependent, thus some environmental factors and personal mood at that time may affect the questionnaire [98].

Comorbidity of chronic pain with psychiatric disorders is common, with prevalence rates of anxiety disorders ranging between 2.3 and 35.1% [105,106,107,108], mood disorders between 6.0 and 28.6% [105,107,109], and substance use disorders between 2.5 and 5.8% [107,110] among individuals with chronic pain in the general population. Chronic pain and depression exhibit a bidirectional relationship, mediated by neuroplasticity that involves similar brain structures, neurotransmitters, and signaling pathways, leading to both psychological and physical symptoms [109,111,112,113,114,115]. Patients with comorbid depression and chronic pain show lower response rates to antidepressants and a higher incidence of suicide attempts than those without chronic pain [116]. Fibromyalgia, a common chronic pain disorder, is often comorbid with depression, leading to heightened pain perception and decreased quality of life [117,118,119]. Furthermore, low serum levels of vitamin D may exacerbate fibromyalgia symptom severity and depression in patients with fibromyalgia [46]. Therefore, vitamin D supplementation may alleviate the psychological and somatic symptoms of fibromyalgia, resulting in improved quality of life for patients.

The issue of “biological flaws” is critical in studies involving vitamin D [17,120]. Biological flaws refer to interventions that do not improve vitamin D status, interventions that do not include vitamin D, lack of baseline vitamin D level, or sufficient vitamin D level at baseline (i.e., patients without vitamin D deficiency) [121]. These biological flaws can result in inconsistent outcomes of vitamin D studies. In this meta-analysis, we carefully assessed the vitamin D levels and presented the data in Supplementary Table S1. Seven out of eight studies included in our analysis did not have any biological flaws. However, one study [99] did not report the mean vitamin D level before and after supplementation, although they included patients with confirmed low levels of vitamin D. This biological flaw may have contributed to some of the inconsistencies observed in our meta-analysis.

Our study has several limitations. First, the sample size in our meta-analysis was small, which precluded us from conducting sensitivity analysis and subgroup analysis. Second, the diagnostic criteria employed across the eight articles included in our study differed, and this may have contributed to heterogeneity among the findings. Third, while the psychological aspect of fibromyalgia is an important component of patients’ quality of life, other confounding factors may also influence our results. Fourth, the trials included in our analysis had short research periods, and therefore, long-term effects of the intervention remain unclear. Fifth, although some evidence suggests a negative correlation between fibromyalgia symptoms and serum vitamin D levels [122,123,124], we did not explore the association between the serum vitamin D level and the severity of fibromyalgia symptoms. Furthermore, vitamin D supplementation cannot be directly understood as the increase of serum vitamin D level.

## 5. Conclusions

Our study demonstrates that vitamin D is a safe, well-tolerable, and alternative intervention for improving psychological outcomes and quality of life in patients with fibromyalgia. However, to confirm the clinical implications of these findings, future trials with larger sample sizes, controlling for potential confounding factors, and with long-term follow-up are warranted.

## Figures and Tables

**Table 1:** Characteristics of the eight included studies.

First Author	Year	Country	Study Design	FMS Diagnostic Criteria	Participants (N)	Age	Dose of Vitamin D	Assessment	Major Findings	Duration
					Tx/Placebo	Mean (SD)				Weeks
Warner [31]	2008	USA	RCT	1990 ACR criteria	22/20	57.4 (9.3)	50,000 IU/week	VAS	No difference in the VAS	12
Arvold [92]	2009	USA	RCT	Not mentioned	48/42	59.7 (14.0)	50,000 IU/week	FIQ	The treatment group showed mild short-term improvement in the overall FIQ	8
Wepner [93]	2014	Austria	RCT	1990 or 2010 ACR criteria	15/15	48.4 (5.3)	50,000 IU/week	VAS, FIQ, SF-36	Treatment group showed marked reduction in VAS, with better physical functioning scale in SF-36	20
Knutsen [96]	2014	Norway	RCT	Not mentioned	144/71	36 (3)	1000 IU/day	VAS	Treatment group showed improved pain scores and headache scores	16
Mirzaei [94]	2018	Iran	RCT	2010 ACR criteria	37/37	42.1 (10.8)	50,000 IU/week	FIQ, SF-36, WPI, PSQI	Improvement in FIQ, WPI, PSQI scores in both groups; (patients of both groups took Trazodone 25 mg before sleep)	8
Lozano-Plata [95]	2020	Mexico	RCT	1990 or 2010 ACR criteria	39/40	50.3 (11.9)	50,000 IU/week	VAS, FIQ	No difference in the VAS or FIQ	12
Yilmaz [97]	2016	Turkey	Prospective	1990 ACR criteria	58/0	36.9 (9.2)	50,000 IU/week	VAS, SF-36, BDI	Reduced in VAS, BDI, improved in some subgroups of SF-36	12
Dogru [98]	2017	Turkey	Prospective	2010 ACR criteria	70/0	38.7 (5.2)	50,000 IU/week	VAS, FIQ, SF-36, BDI	No difference in VAS, FIQ, BDI, improved in overall SF-36	12

Abbreviations: FMS = fibromyalgia; Tx = treatment; SD = standard deviation; RCT = randomized controlled trial; ACR = American College of Rheumatology; VAS = Visual Analogue Scale; FIQ = Fibromyalgia Impact Questionnaire; SF-36 = the 36-item Short Form Health Survey; BDI = Beck Depression Inventory; WPI = Widespread Pain Index; PSQI = Pittsburgh Sleep Quality Index.

## Data Availability

All relevant data were provided in the manuscript.

## Supplementary Materials

The following supporting information can be downloaded at: https://www.mdpi.com/article/10.3390/jcm12072750/s1, Table S1: Baseline and final serum vitamin D level of the included studies. Table S2: Reasons for exclusion of the articles. References [4,5,7,10,14,18,25,29,31,32,33,34,35,36,37,38,39,40,41,42,43,44,45,45,46,47,48,49,50,51,52,53,54,55,56,57,58,59,60,61,62,63,64,65,66,67,68,69,70,71,72,73,74,75,76,77,78,79,80,81,82,83,84,85,86,87,88,89] are shown in Supplementary Table S2.
