# Supplementary material for: Psychological Outcomes and Quality of Life of Fibromyalgia Patients with Vitamin D Supplementation—A Meta-Analysis
Source: J Clin Med. 2023 Apr 6;12(7):2750. doi: 10.3390/jcm12072750 (PMC10095322; doi:10.3390/jcm12072750)
Supplement: Supplementary file 1 [file jcm-12-02750-s001.zip › Supplementary Table S1.pdf]

Supplementary Table S1. Baseline and final serum vitamin D level of the included studies

Author	Year	Country	Study design	Participants (N)	Dose of Vitamin D	Duration	Baseline serum 25 (OH) D level (ng/ml) in treatment group	Baseline serum 25 (OH) D level (ng/ml) in control group	Final serum 25 (OH) D level (ng/ml) in treatment group	Final serum 25 (OH) D level (ng/ml) in control group	Presence of biological flaws
				Tx/ Placebo		Weeks	Mean ± SD	Mean ± SD	Mean ± SD	Mean ± SD	N/Y
Warner	2008	USA	RCT	22/20	50000 IU/ week	12	16.8 ± 2.9	15.9 ± 3.6	31.2 ± 6.2	19.3 ± 6.5	N
Arvold	2009	USA	RCT	48/42	50000 IU/ week	8	17.9 ± 3.5	18.1 ± 4.0	45 ± 12	21.5 ± 5.5	N
Wepner	2014	Austria	RCT	15/15	50000 IU/ week	20	19 ± 5.91	20.89 ± 6.27	51 ± 20.6	28.2 ± 12.4	N
Knutsen	2014	Norway	RCT	144/71	1000 IU/ day	16	10.8 ± 6	10.8 ± 6	20.8 ± 8.8	10 ± 4.8	N
Yilmaz	2016	Turkey	Prospective	58/0	50000 IU/ week	12	10.6 ± 5.1	NA	46.5 ± 24	NA	N
Dogru	2017	Turkey	Prospective	70/0	50000 IU/ week	12	NA	NA	NA	NA	Y
Mirzaei	2018	Iran	RCT	37/37	50000 IU/ week	8	11.4 ± 6.5	13.4 ± 7.3	33.5 ± 12.2	13.3 ± 7.2	N
Lozano-Plata	2020	México	RCT	39/40	50000 IU/ week	12	20.1 ± 14.5	12.6 ± 13.4	51.1 ± 16.5	20.8 ± 7.9	N

Abbreviations: Tx= Treatment; SD= Standard Deviation; RCT= Randomized controlled trial; N= No, Y= Yes
