# Supplementary material for: Psychological Outcomes and Quality of Life of Fibromyalgia Patients with Vitamin D Supplementation—A Meta-Analysis
Source: J Clin Med. 2023 Apr 6;12(7):2750. doi: 10.3390/jcm12072750 (PMC10095322; doi:10.3390/jcm12072750)
Supplement: Supplementary file 1 [file jcm-12-02750-s001.zip › Supplementary Table S2.pdf]

Supplementary Table S2. Reasons for exclusion of the articles

First Author / Publication Year	Journal	Title	Reason for exclusion
Abou-Raya et al., 2014	Annals of the Rheumatic Diseases	Efficacy of vitamin D supplementation in the treatment of fibromyalgia: Randomized controlled trial	Meeting Abstract
Al-Allaf et al., 2002	Journal of Bone and Mineral Research	Fibromyalgia, bone density and vitamin D deficiency	Not RCT/cohort study
Al-Allaf et al., 2003	Annals of the Rheumatic Diseases	Fibromyalgia, bone density and vitamin D deficiency	Meeting Abstract
Al-Dabbagh et al., 2012	Saudi Medical Journal	The relation between vitamin D deficiency and fibromyalgia syndrome	Editorial
Aldaoseri et al., 2020	Egyptian Rheumatologist	Vitamin D deficiency and treatment in Iraqi patients with primary fibromyalgia syndrome	No VAS, FIQ, BDI, SF-36 scores
Ali et al., 2022	SN Comprehensive Clinical Medicine	Prevalence of Vitamin D Deficiency and Its Relationship with Clinical Outcomes in Patients with Fibromyalgia: A Systematic Review of the Literature	Review article
Balkarli et al., 2016	Annals of the Rheumatic Diseases	Haplotypes of vitamin d receptor gene: Both risky and protective for fibromyalgia (FMS)	Gene study
Block et al., 2004	Mayo Clinic Proceedings	Vitamin D deficiency is not associated with nonspecific musculoskeletal pain syndromes including fibromyalgia	Letter to editor
Braun et al., 2004	Arthritis and Rheumatism	Fibromyalgia and vitamin D deficiency: Is there a correlation	Meeting abstract
Carvalho et al., 2018	Isr Med Assoc J	Vitamin D Supplementation Seems to Improve Fibromyalgia Symptoms: Preliminary Results	No VAS, FIQ, BDI, SF-36 scores
Cassisi et al., 2013	Clinical and Experimental Rheumatology	Complementary and alternative medicine in fibromyalgia: A practical clinical debate of agreements and contrasts	Review article
Daniel et al., 2011	Aust Fam Physician	Fibromyalgia--should we be testing and treating for vitamin D deficiency?	Review article
Dogru et al., 2016	Annals of the Rheumatic Diseases	Effects of vitamin D therapy on quality of life in patients with fibromyalgia	Meeting abstract

Dogru et al., 2016	Annals of the Rheumatic Diseases	Effects of vitamin d therapy on quality of life in patients with fibromyalgia	Poster abstract
D'Souza et al., 2020	Pain Med	Fibromyalgia Symptom Severity and Psychosocial Outcomes in Fibromyalgia Patients with Hypovitaminosis D: A Prospective Questionnaire Study	No VAS, FIQ, BDI, SF-36 scores
Ellis et al., 2018	BMC Rheumatology	The role of vitamin D testing and replacement in fibromyalgia: A systematic literature review	Review article
Hsiao et al., 2015	Pain Physician	Is serum hypovitaminosis D associated with chronic widespread pain including fibromyalgia? A meta-analysis of observational studies	Review article
Huisman et al., 2000	Arthritis and Rheumatism	Vitamin D in female systemic lupus erythematosus and fibromyalgia patients	Meeting abstract
Ibáñez-Vera et al., 2018	Apunts Medicina de l'Esport	Therapeutic physical exercise and supplements to treat fibromyalgia	Review article
Jesus et al., 2013	Current Pain and Headache Reports	The Role of Vitamin D in Pathophysiology and Treatment of Fibromyalgia	Review article
Kadayifci et al., 2022	Nutrition Reviews	Review of nutritional approaches to fibromyalgia	Review article
Karkeni et al., 2018	Epigenetics	Vitamin D limits inflammation-linked microRNA expression in adipocytes in vitro and in vivo: A new mechanism for the regulation of inflammation by vitamin D	Gene study
Karras et al., 2016	Nutrients	Vitamin D in fibromyalgia: A causative or confounding biological interplay?	Review article
Khan et al., 2016	Rheumatology	Early replacement of vitamin D in vitamin D-deficient fibromyalgia patients improves reported pain and number of tender points	Meeting abstract
Kvæl et al., 2021	Nutrients	Use of Dietary Supplements and Perceived Knowledge among Adults Living with Fibromyalgia in Norway: A Cross-Sectional Study	No VAS, FIQ, BDI, SF-36 scores
Lewis et al., 2011	Journal of Musculoskeletal Pain	Fibromyalgia syndrome and Vitamin D	Review article
Lombardo et al.,2022	Nutrients	The Efficacy of Vitamin D Supplementation in the Treatment of Fibromyalgia Syndrome and Chronic Musculoskeletal Pain	Review article
Ma et al., 2017	DNA and Cell Biology	Vitamin D supplementation prevents placental ischemia induced endothelial dysfunction by downregulating placental soluble FMS-like tyrosine kinase-1	Animal study

Makrani et al., 2017	Korean Journal of Pain	Vitamin D and fibromyalgia: A meta-analysis	Review article
Maraslı et al., 2016	International Journal of Rheumatic Diseases	Relationship between Fokl polymorphism in the vitamin D receptor gene and fibromyalgia syndrome	Gene study
Martin et al., 1999	Journal of Musculoskeletal Pain	Potential clinical benefit of nutritional supplements in the management of fibromyalgia	Review article
Martins et al., 2020	Nutrition Reviews	Is hypovitaminosis D associated with fibromyalgia? A systematic review	Review article
Matthana et al., 2011	Saudi Medical Journal	The relation between vitamin D deficiency and fibromyalgia syndrome in women	No VAS, FIQ, BDI, SF-36 scores
Matthana et al., 2012	Saudi Medical Journal	The relation between vitamin D deficiency and fibromyalgia syndrome Reply	Letter to editor
Miao et al., 2008	Medical Acupuncture	How do you treat fibromyalgia in your practice?	Editorial
Moreno García et al., 2017	Revista de la Sociedad Espanola del Dolor	Fibromyalgia and vitamin D levels	Editorial
Motley et al., 2010	Primary Care - Clinics in Office Practice	Fibromyalgia: Helping your patient while maintaining your sanity	Review article
Mytton et al., 2007	British Journal of General Practice	Vitamin D deficiency in multicultural primary care: A case series of 299 patients	Case series
Nct et al., 2017	Clinicaltrials.gov	Valuation of Efficacy and Safety of Vitamin D3 Use in 80 Women Diagnosed With Fibromyalgia. Using FIQ Score and VAS	Not RCT/cohort study
Padidela et al., 2020	Orphanet J Rare Dis	The international X-linked hypophosphataemia (XLH) registry (NCT03193476): rationale for and description of an international, observational study	Review article
Pagliai et al., 2020	Nutrients	Nutritional interventions in the management of fibromyalgia syndrome	Review article
Parvez et al., 2022	Cureus	Relationship Between Vitamin D Receptor Gene BsmI Polymorphism and Fibromyalgia Syndrome	Gene study
Plotnikoff et al., 2004	Mayo Clinic Proceedings	Vitamin D deficiency is not associated with nonspecific musculoskeletal pain syndromes including fibromyalgia - Reply	Letter to editor

Qu et al., 2022	Expert Review of Clinical Pharmacology	The efficacy of vitamin D in treatment of fibromyalgia: a meta-analysis of randomized controlled studies and systematic review	Review article
Rivera et al., 2009	Clin Exp Rheumatol	Resource utilisation and health care costs in patients diagnosed with fibromyalgia in Spain	No VAS, FIQ, BDI, SF-36 scores
Rossi et al., 2015	Clinical and Experimental Rheumatology	Fibromyalgia and nutrition: what news?	Review article
Scheuer et al., 2014	International Journal of Clinical Rheumatology	Fibromyalgia: Could vitamin D help?	Review article
Shanmuganandn et al., 2012	Indian Journal of Rheumatology	Vitamin D deficiency: A 'Blindspot' for clinicians and a veritable 'Ray' of hope for patients with chronic musculoskeletal pains?	Review article
Sicras-Mainar et al., 2009	Arthritis Research & Therapy	Treating patients with fibromyalgia in primary care settings under routine medical practice: a claim database cost and burden of illness study	No VAS, FIQ, BDI, SF-36 scores
Siracusa et al., 2021	International Journal of Molecular Sciences	Fibromyalgia: Pathogenesis, mechanisms, diagnosis and treatment options update	Review article
Sousa et al., 2022	Annals of the Rheumatic Diseases	VITAMIN D SUPPLEMENTATION in FIBROMYALGIA: A SYSTEMATIC REVIEW	Review article
Spaeth et al., 2007	Journal of Musculoskeletal Pain	Fibromyalgia syndrome review	Review article
Straube et al., 2009	Pain	Vitamin D and chronic pain	Review article
Straube et al., 2015	Cochrane Database of Systematic Reviews	Vitamin D for the treatment of chronic painful conditions in adults	Review article
Talotta et al., 2017	Clinical and Experimental Rheumatology	One year in review 2017: Fibromyalgia	Review article
Türkyilmaz et al., 2010	Osteoporoz Dunyasindan	The effects of bone mineral density and level of serum vitamin-D on pain and quality of life in fibromyalgia patients	Poster abstract
Vasquez et al., 2016	Nature Reviews Rheumatology	Neuroinflammation in fibromyalgia and CRPS is multifactorial	Review article

Velozo et al., 2008	Bone	Vitamin D levels on patients with Fibromyalgia	Not RCT/cohort study
Venkatesan et al., 2022	Current rheumatology reviews	Efficacy of Vitamin D Supplementation in the Improvement of Clinical Status in Patients Diagnosed with Fibromyalgia Syndrome: A Systematic Review	Review article
Vittorio et al., 2020	Nutrients	Comparison between Acupuncture and Nutraceutical Treatment with Migratens (R) in Patients with Fibromyalgia Syndrome: A Prospective Randomized Clinical Trial	No VAS, FIQ, BDI, SF-36 scores
Walitt et al., 2011	J Rheumatol	The longitudinal outcome of fibromyalgia: a study of 1555 patients	No VAS, FIQ, BDI, SF-36 scores
Warner et al., 2005	Arthritis and Rheumatism	Treatment with vitamin D does not reduce pain in fibromyalgia patients with low vitamin D levels	Not RCT/cohort study
Warner et al., 2008	Journal of Clinical Rheumatology	Diffuse musculoskeletal pain is not associated with low vitamin D levels or improved by treatment with vitamin D	No VAS, FIQ, BDI, SF-36 scores
Wu et al., 2016	Pain Physician	Effect of Vitamin D Supplementation on Pain: A Systematic Review and Meta-analysis	Review article
Wu et al., 2018	Public Health Nutrition	The association between vitamin D concentration and pain: a systematic review and meta-analysis	Review article
Yong et al., 2017	Clinical Rheumatology	Effect of vitamin D supplementation in chronic widespread pain: a systematic review and meta-analysis	Review article
Zabihiyeganeh et al., 2015	Osteoporosis International	Effects of vitamin D on quality of life in fibromyalgia patients: A randomized controlled trial	Poster abstract
Zdebik et al., 2021	Survey of Ophthalmology	Fibromyalgia syndrome and the eye-A review	Review article
